# Identification of Anti-tuberculosis Compounds From Aurone Analogs

**DOI:** 10.3389/fmicb.2020.01004

**Published:** 2020-05-20

**Authors:** Dong Yang, Zachary E. Taylor, Scott Handy, Shaoji Li, Jiawang Liu, Jennifer Stabenow, Lillian Zalduondo, Colleen B. Jonsson, Elliot Altman, Ying Kong

**Affiliations:** ^1^Department of Microbiology, Immunology, and Biochemistry, University of Tennessee Health Science Center, Memphis, TN, United States; ^2^Department of Chemistry, Middle Tennessee State University, Murfreesboro, TN, United States; ^3^Tennessee Center for Botanical Medicine Research, Middle Tennessee State University, Murfreesboro, TN, United States; ^4^Medicinal Chemistry Core, University of Tennessee Health Science Center, Memphis, TN, United States; ^5^Regional Biocontainment Laboratory, University of Tennessee Health Science Center, Memphis, TN, United States; ^6^Department of Biology, Middle Tennessee State University, Murfreesboro, TN, United States

**Keywords:** tuberculosis, *Mycobacterium tuberculosis*, aurone analogs, chorismate synthase, anti-TB agents

## Abstract

The emergence of multidrug-resistant *Mycobacterium tuberculosis* (*Mtb*) strains has made tuberculosis (TB) control more difficult. Aurone derivatives have demonstrated promising anti-bacterial activities, but their effects against *Mtb* have not been thoroughly determined. In this study, we aimed to develop anti-TB compounds from aurone analogs. We used a fluorescent protein tdTomato labeled *Mtb* CDC1551 strain to screen 146 synthesized aurone derivatives for effective anti-TB compounds. The 9504, 9505, 9501, 9510, AA2A, and AA8 aurones inhibited the growth of *Mtb* with minimal inhibitory concentrations of 6.25, 12.5, 25, 25, 25, and 50 μM, respectively. We also examined cytotoxicities of the six leads against the human liver cell line HepG2, the primate kidney cell line Vero and human monocyte THP-1 derived macrophages. Three of the aurone leads (9504, 9501, and 9510) showed low cytotoxic effects on all three cell lines and high *Mtb* inhibitory efficacy (selectivity index > 10). Aurone 9504, 9501, AA2A, or AA8 significantly reduced the *Mtb* load in the lungs of infected mice after a 12-days treatment. We determined that the aurone leads inhibit *Mtb* chorismate synthase, an essential enzyme for aromatic acid synthesis. Our studies demonstrate the promise of synthetic aurones as novel anti-TB therapeutics.

## Introduction

Tuberculosis (TB) ranks as one of the leading causes of death worldwide due to a bacterial pathogen. In particular, pulmonary TB is a highly contagious and life-threatening infection (WHO, 2018). The recommended anti-TB regimen is a combination of at least four drugs for at least 6 months, and up to 2 years for drug-resistant TB (WHO, 2004; Caminero et al., 2010), which often results in incomplete treatment regimens (Ginsberg and Spigelman, 2007). During the past decade, incidences of multidrug-resistant TB (MDR-TB) and extensively drug-resistant TB (XDR-TB) have been increasing in both developing and industrialized countries (WHO, 2018). These situations underscore the importance of the development of new anti-TB drugs. Simpler drug regimens that are safe, well tolerated, and effective against MDR/XDR TB are urgently needed. Although considerable progress has been made in recent years to put novel anti-TB agents into the drug development pipeline, the number and efficacies of new anti-TB drugs have not fully met therapeutic needs (WGNTD, 2016).

Aurones are naturally occurring compounds, responsible for the yellow pigments in vegetables and flowers, and are structural isomers of flavones (Harborne, 1988). Although the studies of the biological activities of aurones are still limited, synthetic aurones have been reported to be promising bioactive compounds, effectively inhibiting the growth of bacteria, including *Staphylococcus aureus* and *Caulobacter crescentus* (Pires et al., 2001), *Streptococcus pneumoniae* (Thomas et al., 2003), *Klebsiella pneumoniae* (Hadj-esfandiari et al., 2007), *Bacillus subtilis*, *E. coli*, *Proteus vulgaris*, and fungi (Bandgar et al., 2010; Sutton et al., 2017). However, the anti-TB activities of these compounds have not been carefully investigated so far, with only a single broad-spectrum kinase inhibitor screening that included a few aurones having been reported (Reynolds et al., 2012).

We have used a fluorescent-protein labeled *Mycobacterium tuberculosis* (*Mtb*) CDC1551 strain (Kong et al., 2016) to screen for effective anti-TB aurone derivatives, because this approach is more time-efficient than conventional colony forming unit (CFU) enumeration on agar plates (Barer and Harwood, 1999; Zhang, 2004). We successfully screened 146 aurone analogs using this *Mtb* strain and identified six aurone derivatives, designated as 9504, 9505, 9501, 9510, AA2A, and AA8, that have significantly inhibitory/eliminatory effects against *Mtb* growth *in vitro.* We determined the cytotoxic effects of these six aurones against the human liver cell line HepG2, the primate kidney cell line Vero, and the human monocyte derived macrophage THP-1 cells. We also evaluated their efficacies against intracellular *Mtb* in the THP-1 cell derived macrophage and determined *in vivo* efficacies of the four most promising aurone leads (9504, 9501, AA2A, and AA8) in BALB/c mice. Furthermore, we demonstrated that the aurone leads can inhibit chorismate synthase, the key enzyme of the shikimate pathway.

## Materials and Methods

### Aurone Synthesis

Aurones were synthesized using either the method described by Varma and Varma (1992) or the method reported by Hawkins and Handy (2013). The azaaurones were synthesized via a modification of the method reported by Carrasco et al. (2016). To a solution of 1-acetylindolin-3-one (0.5 mmol) in toluene (3 mL), the appropriate aldehyde (0.5 mmol) and 1 drop of piperidine was added. The mixture was heated to reflux for 12 h, cooled to room temperature, and then purified by flash column chromatography using an ethyl acetate/hexanes mixture. For deacetylated azaaurones, the acetylated product was dissolved in methanol (2 mL) and treated with 0.1 mL of 50% aqueous KOH for 45 min. The reaction mixture was acidified and extracted with ethyl acetate and concentrated *in vacuo*. The resulting residue was purified via flash column chromatography using toluene/ethanol mixtures to afford the desired azaaurones.

#### AA2A

To a solution of 0.5 mmol of 1-acetylindolin-3-one in 1 mL of toluene, a slight excess (1.2 equivalents) of 2-bromobenzaldehyde and one drop of piperidine was added. The mixture was heated to 100°C for 12 h, then cooled to room temperature and purified via flash column chromatography (20% ethyl acetate/hexanes) to afford the desired compound as a yellow solid (mp = > 200°C) in 83% yield. ^1^H NMR (300 MHz, CDCl_3_) 8.28 (d, J = 8 Hz, 1H, H4), 7.87 (d, J = 8 Hz, 1H, H7), 7.71-7.63 (m, 2H, H2′, 4′), 7.46 (dd, J = 1.5, 8 Hz, 1H, H5′), 7.42 (s, 1H, = CH), 7.35 (t, J = 8 Hz, 1H, H5), 7.32 (d, J = 8 Hz, 1H, H6′), 7.24 (t, J = 8 Hz, 1H, H6), 1.81 (s, 3H, CH_3_); ^13^C NMR (75 MHz, CDCl_3_) 185.42, 170.08, 150.27, 136.70, 136.30, 135. 75, 133.70, 130.82, 130.26, 128.00, 125.32, 125.14, 124.45, 123.94, 120.76, 117.82, 24.80.

#### AA8A

To a solution of 0.5 mmol of 1-acetylindolin-3-one in 1 mL of toluene, a slight excess (1.2 equivalents) of benzaldehyde and one drop of piperidine was added. The mixture was heated to 100°C for 12 h, then cooled to room temperature and purified via flash column chromatography (20% ethyl acetate/hexanes) to afford the desired compound as a yellow oil that was mostly the E isomer (>5:1) in 80% yield. ^1^H NMR (300 MHz, CDCl_3_) 8.30 (d, J = 8 Hz, 1H, H4), 7.86 (d, J = 8 Hz, 1H, H7), 7.71-7.62 (m, 1H, H4′), 7.55 (d, J = 8 Hz, 2H, H2′, 6′), 7.45-7.35 (m, 2H, H3′, 5′), 7.35 (t, J = 8 Hz, 1H, H5), 7.33 (s, 1H, = CH), 7.29 (t, J = 8 Hz, 1H, H6), 1.92 (s, 3H, CH_3_); ^13^C NMR (75 MHz, CDCl_3_) 186.02, 170.55, 150.36, 136.48, 135.12, 134.12, 130.29 (2C), 129.98, 129.32 (2C), 125.04, 124.24, 123.99, 122.42, 117.94, 25.19.

#### AA8

To a solution of AA8A in 2 mL of methanol, 0.4 mL of 50% aqueous KOH was added. After stirring for 30 min, the reaction was neutralized with 1M HCl, extracted with ethyl acetate, and the organic layer dried with sodium sulfate, filtered and concentrated *in vacuo*. The resulting residue was purified via flash column chromatography (20% ethanol in toluene) to afford the desired compound as an orange solid (mp = 189–191°C) in 80% yield. ^1^H NMR (300 MHz, CDCl_3_): 7.76 (d, J = 8 Hz, 1H, H4), 7.56 (d, J = 8 Hz, 2H, H2′, 6′), 7.51-7.42 (m, 3H, H5, 7, 4′), 7.33 (t, J = 8 Hz, 1H, H6), 7.03-6.98 (m, 2H, H3′, 5′), 6.88 (s, 1H, = CH); ^13^C NMR (75 MHz, CDCl_3_): 186.72, 153.29, 136.37, 135.54, 134.89, 129.63, 128.74, 125.19, 121. 88, 120.82, 112.10, 111.67.

### *Mtb* Strains and Culture

The *Mtb* CDC1551 strain was grown in 7H9 broth (Difco, Detroit, MI) supplemented with 0.5% glycerol, 10% OAD (oleic acid dextrose complex without catalase) and 0.05% Tween 80 (M-OAD-Tw broth), or Middlebrook 7H9 supplemented with 10% OAD and 15 g/L Bacto agar (M-OAD agar, Difco), or on 7H11 selective agar (Difco). The media were kept in the dark to avoid accumulation of hydrogen peroxide, and thus the addition of catalase in the media was not required. Previously, we have constructed the plasmid expressing tdTomato under the mycobacterial phage L5 promoter (Kong et al., 2016). In brief, we first PCR amplified the *tdTomato* gene from pRSETB-tdTomato using an up-stream primer containing a *Hind*III site at the 5′ end and a down-stream primer containing *Kpn*I site at the 5′ end. We then cut pFJS8, an *E. coli-*mycobacterium shuttle plasmid containing the L5 promoter (Miltner et al., 2005), with *Hind*III / *Kpn*I, and separated the larger fragment by gel purification. The *Hind*III / *Kpn*I digested PCR products were also gel purified to obtain the tdTomato specific fragment, which was ligated into the *Hind*III / *Kpn*I cut pFJS8 linear fragment, to form pJDC60 (L5-tdTomato). This plasmid was then electroporated into the CDC1551 competent cells and plated on hygromycin selective 7H10 agar plates to identify the clone of tdTomato expressing CDC1551. To culture the tdTomato-expressing *Mtb* CDC1551 strain, media and plates were supplemented with 80 μg/mL hygromycin. Frozen stocks were prepared from strains by growth without shaking at 37°C until an OD_600_ = 0.5 was reached, and then stored in aliquots at –80°C until use.

### Minimum Inhibitory Concentrations (MICs) of Aurones

The standard resazurin microtiter assay was used to determine MICs of the six aurone leads. Black 96-well microplates were preloaded with 100 μL of two-fold serial dilutions of aurones (1.56–100 μM) or rifampicin (RIF) (0.0625–4 μM) in M-OAD-Tw with 3 replicates per concentration. After adjusting the absorbance of the bacterial culture to a McFarland tube no. 1, the bacteria were diluted 1:20 with the medium, and 100 μL was used as an inoculum to load into each well. The plates were covered, sealed in plastic bags, and incubated at 37°C in normal atmosphere. After 7 days of incubation, 30 μL of resazurin solution (0.02%) was added to each well, incubated overnight at 37°C, and assessed for color development. A change from blue to pink indicates reduction of resazurin and therefore bacterial growth. All MICs were performed in duplicate from at least two independent cultures.

### Cytotoxicity Assay

Cytotoxicities of the aurones were evaluated in Vero cells (ATCC), HepG2 (ATCC), and THP-1 cells (ATCC) using the standard microculture tetrazolium (MTT) cell viability assay. Media used for the Vero cells was DMEM supplemented with 10% calf serum; for HepG2 cells the media used was DMEM supplemented with 20% calf serum; and for THP-1 cells the media used was RPMI-1640 supplemented with 10% calf serum (HyClone Cosmic Calf, GE Lifesciences). Cells were seeded into a 96-well plate. For THP-1 cells, phorbol-12-myristate-13-acetate (PMA) was used to induce the monocytes to develop into macrophages for 3 days before the cytotoxicity assay. A series of two-fold dilutions of aurones or RIF were added into the cell culture media in microplates to determine the concentration that eliminated 50% of eukaryotic cell growth in a 2-days incubation. Cells without drug treatment were incubated with the vehicle buffer and served as negative controls (100% viable). Cells treated with digitonin (100 μg/mL) served as the positive controls. After a 2-days incubation, 10 μL of MTT reagent was loaded into each well, and incubated for 3 h at 37°C. Hundred microliter of detergent solution was then added into each well and the 96-well plates were incubated for 2 h. Absorbance of each sample in the 96-well plates was measured at 580 nM. Viability of each drug-treated sample was calculated as the absorbance of the sample divided by the absorbance of the untreated sample multiplied by 100.

### Efficacies of Aurones Against Intracellular *Mtb* Infection

Human monocyte THP-1 cells were seeded at 5 × 10^4^ cells / well in 96-well tissue culture black plates with clear bottoms. PMA was used to induce the monocytes to develop into macrophages for 3 days before infection. The tdTomato-expressing *Mtb* strain was employed for cell infection at an MOI = 20. After a 3 h incubation of the *Mtb* strain with THP-1 cells, extracellular bacteria were removed by washing twice with the cell culture medium. 200 μL of cell culture medium containing either 25 or 50 μM aurones or 1X MICs of amikacin (AMI, 1 μg/mL), ethambutol (ETH, 0.5 μg/mL), RIF (0.4 μg/mL) and isoniazid (INH) (0.5 μg/mL) was added to each well in triplicate and incubated for 48 h. Infected cells treated with the vehicle buffer served as negative controls. Cells were then lysed and plated for CFU counting.

### Potencies of Aurones Against *Mtb* Infection in Mice

Female BALB/c mice (5–7 weeks old) were obtained from Jackson Laboratories. All animals were housed in the UTHSC Regional Biocontainment Laboratory (RBL) in a controlled environment with 12 h light/12 h dark cycle, ∼18–23°C, and 40–60% humidity.

#### Pilot Drug-Tolerance-Test

Prior to testing *in vivo* efficacies of the aurones, we determined the drug-tolerance of the aurones in BALB/c mice using a single dose range-finding study. AA2A, AA8, 9501, and 9504 stock solutions (40 mg/mL) dissolved in DMSO were mixed 1:1 with Tween 80 to improve aqueous solubility, and then added into phosphate buffer to four final concentrations for delivering 1, 2, 5, 10 mg/kg of the aurones into mice. In mice, intraperitoneal (i.p.) injection is used predominantly for the administration of systemic drugs and fluids because of the ease of administration compared with intravenous injection. Therefore, we i.p. injected aurones into BALB/c mice daily for 7 days. Mice were weighed daily and examined twice daily for adverse effects by veterinarians and specialized technicians.

#### Mouse Infection and Aurone Treatment

BALB/c mice were infected using the Bio-Aerosol Nebulizing Generator in UTHSC RBL, with 5 × 10^5^ cfu/mL of *Mtb* in PBS to deliver ∼10–20 cfu/lung. At day-28 post-infection, mice were randomly grouped into the vehicle-treated, AA2A-, or AA8-, or 9501-, or 9504-treated groups (6 mice/group/time point). For the aurone-treated groups, 5 mg/kg of AA2A, AA8, 9504, or 9501 was injected via i.p. daily. The control group of mice were injected with the vehicle buffer (10 mM phosphate buffer + 2.5% Tween 80 + 2.5% DMSO). The IVIS Spectrum *in vivo* imaging system (PerkinElmer) was used to collect *ex vivo* images for the harvested lungs 1-day before the treatment and on day-12 post-treatment, following the protocol described previously by Kong et al. (2016). Mice were euthanized by inhalation of an overdose of isoflurane (>5%), followed by cervical dislocation at the designed time points. Bacterial CFU counts in the lungs of each group of mice were also collected by plating homogenized lungs on 7H11 agar plates at days-1, -21, -28, and -40.

### *Mtb* Chorismate Synthase Activity Assay

The ability of the aurones to inhibit *Mtb*-Cs was determined using a coupled enzyme reaction. *Mtb*-Cs needs *Mtb* EPSPs for biosynthesis of EPSP, which is the substrate for Cs, and *Mtb*-Cs for the formation of chorismate.

#### Expression and Purification of *Mtb* 5-Enol-Pyruvyl Shikimate-3-Phosphate (EPSP) Synthase and Chorismate Synthase (Cs)

*Mtb* EPSP synthase and Cs were over-expressed in *Escherichia coli* using pET28a(+) and purified from the soluble components of the lysates by affinity chromatography on a HisTrap column via elution with a series of concentrations of imidazole. PCR amplified EPSP (Rv3227) and Cs (Rv2540c) coding sequences were cloned into pET28a(+). PCR was conducted using the primers Rv2540c-F tatacatatggtgttgcgctggatcacc and Rv2540c-R: tataggatccttaaccggagacccgc to amplify Rv2540c from genomic DNA of *Mtb* CDC1551 with the following program: denaturation for 5 min at 95°C, then 34 cycles consisting of 45 sat 95°C, 45 s at the annealing temperature (56°C), and 3 min at 72°C, and then 10 min at 72°C for final extension. To amplify Rv3227 from genomic DNA of *Mtb* CDC1551, PCR was conducted using the primers Rv3227-F: tatacatatggtgaagacatggccagcc and Rv3227-R: tataggatccactcgtcgtagtcgccgg with the following program: denaturation for 5 min at 95°C, then 34 cycles consisting of 45 s at 95°C, 45 s at the annealing temperature (62°C), and 3 min at 72°C, and then 10 min at 72°C for final extension. Further PCR-amplified products of Rv2540c and Rv3227 were analyzed on 0.8% agarose gel and purified with the DNA clean & concentrator kit (Zymo research). Eluted purified PCR products and expression vector pET-28a(+) were digested with *Nde*I and *BamH*I, respectively. The pET-28a(+) vector was also dephosphorylated by phosphatase. After electrophoresis, DNA bands were excised, and then Rv2540c, Rv3227, and the pET-28a(+) vector were extracted using an Agarose GelExtract Mini Kit (5-PRIME). The digested fragments of Rv2540c and Rv3227 were mixed with the pET-28a(+) vector, respectively, and then ligated with T4 DNA ligase. The ligated product was transformed into chemically competent *E. coli* DH5α cells and clones were selected on LB agar plates containing 25 μg/mL kanamycin. The cloned Rv2540c and Rv3227 were confirmed by sequencing (Eurofins Genomics). For expression of Rv2540c and Rv3227, the constructed plasmids were isolated and transformed into expression Rosetta (DE3) competent cells (Novagen). Positive transformants were screened on LB agar plates containing 25 μg/mL kanamycin and confirmed by sequencing. The *E. coli* Rosetta (DE3) strain carrying plasmid *Mtb*-Cs (Rv2540c) or EPSPs (Rv3227) was induced by IPTG over night at 16°C. After lysis of the *E. coli* Rosetta (DE3) cells, the rRv2540c and rRv3227 expressed as His-tagged fusion proteins were purified from the soluble components of the lysates by affinity chromatography on a HisTrap HP Ni^2+^ IMAC column eluted with a series of concentrations of imidazole. The samples were analyzed by SDS-PAGE to confirm the size and purity of the proteins.

#### EPSP Synthesis

The synthesis of EPSP was carried out in a vial containing EPSPs (0.7U), shikimate (9.6 mM), and phosphoenolpyruvate (PEP; 3 mM) at 25°C for 30 min. The equilibrium of the forward reaction was displaced using purine nucleoside phosphorylase (PNP, 2U) and 2-amino-6-mercapto-7-methylpurine ribonucleoside (MESG, 0.4 mM), which consumes phosphate (Pi), increasing the final concentration of EPSP in the reaction mixture. Cleaved MESG results in an absorbance change from 330 to 360 nM. Thus, EPSP synthesis was monitored by measuring A_360_ using a spectrophotometer.

#### Measuring *Mtb*-Cs Activity

After EPSP synthesis, the enzymes were removed by ultrafiltration (3 kD cut-off Centricon), and the filtrate was directly used as a source of EPSP for the chorismate synthesis reaction. The reaction of converting EPSP to chorismate and Pi by Cs contained *Mtb*-Cs, EPSP (15 μL), FMN (0.04 mM), and NADH (0.3 mM). The production of chorismate from the enzymatic reaction was determined by measuring Pi production using MESG (0.2 mM) and PNP (1 U). To assess the aurones effect on Cs, Cs was incubated with various concentrations of the aurone leads in a vial preloaded with EPSP and FMN. After adding NADH into the vial, samples were added into a 96-well plate and mixed with MESG and PNP. The production of chorismate from the enzymatic reaction was indirectly measured by measuring Pi production at A_360_ from the reaction using MESG and PNP. The untreated sample was added to the aurone dilution buffer containing the same concentration of DMSO as those of the aurone-treated samples and served as a positive control (activity 100%). The negative control consisted of the same components as the positive control except that *Mtb* Cs was replaced with the buffer. The absorbance of the negative control was subtracted from the absorbance of the other samples in data analysis. The enzymatic velocity was calculated as the variation of absorbance per minute.

### Determination of EPSP With LC/MS

LC/MS assays were performed to detect EPSP after the EPSP synthesis reaction on a Bruker amaZon SL mass spectrometer (ESI) with a Shimadzu HPLC system equipped with a Kinetex^®^ 5 μm C18 100 Å column (50 × 2.1 mm). A mixture of methanol/water (75:25, v/v) was employed as the major solvent system. The elution started from 100% water for 0.5 min, followed by 75% methanol for 5 min before increasing to 100% methanol over 1 min. The total elution of each injection was 6.5 min at a flow rate of 0.3 mL/min. The MS detector began to record the ion signals at 0.35 min, and both positive and negative ions were collected. It was found that EPSP was eluted out at 1.3 min of the retention time, and the characteristic peak of EPSP was ESI m/z 323.2 [M-1]- under the negative ESI mode. The peak areas (AUC) of EPSP were calculated from extracted ion chromatograms (EIC) of ESI- m/z 323.2 ± 0.5.

### Ethics Statement

The UTHSC Institutional Animal Care and Use Committee (IACUC) approved the animal care and use protocol 16-102 for all animal experiments in this study. UTHSC IACUC adheres to the Public Health Service Policy and Animal Welfare Act.

### Statistical Analysis

Significant difference of means between two groups was examined using the *t*-test. For comparison amongst three or more groups, we employed the ANOVA *F*-test and the Tukey–Kramer method *post-hoc* pairwise *t*-test. The estimation of minimum number of animals for the *in vivo* studies is based on power analysis with the G^∗^Power 3.1 software using conditions based on our previous studies with similar methods (Robertson et al., 2017) at α = 0.05 and Power = 0.8.

## Results

### Six Aurone Leads Were Identified by Structure Activity Relationship (SAR) Studies

**FIGURE 1:**
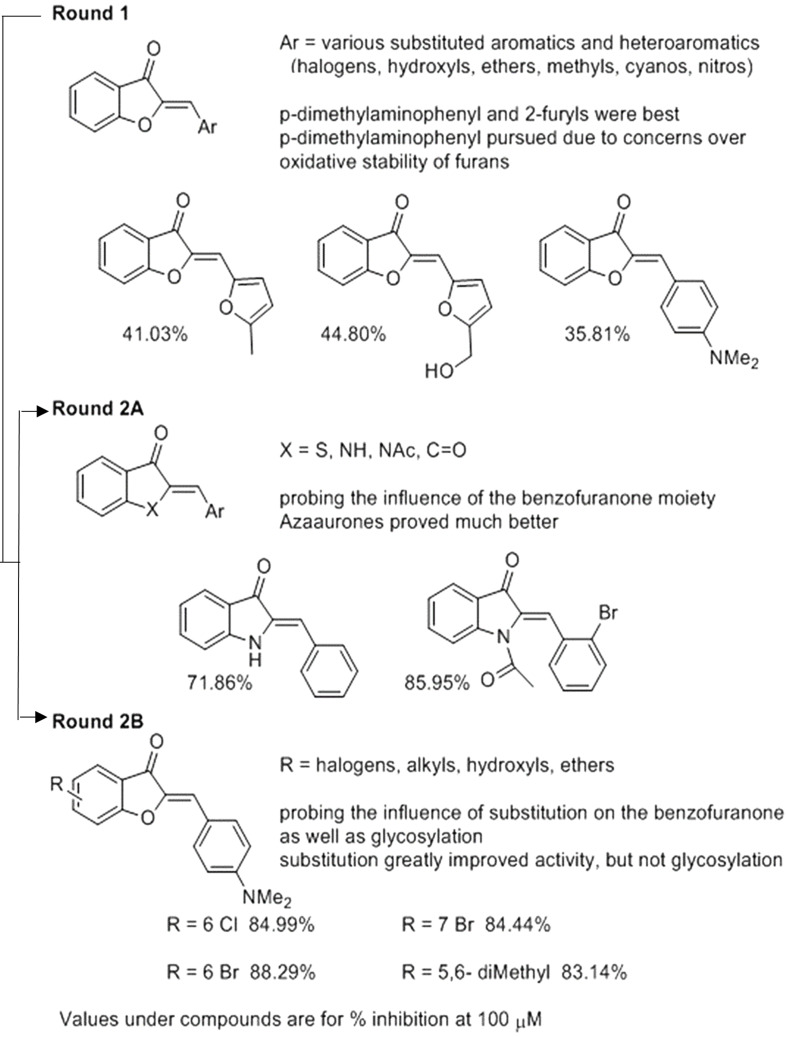
SAR analysis of 146 aurones against *Mtb.*

We used the tdTomato-expressing *Mtb* strain, which can be employed to quantify *Mtb* by directly measuring bacterial fluorescence (Kong et al., 2016), to screen for anti-TB aurone derivatives. In the initial screening, the tdTomato-expressing *Mtb* strain was co-incubated with the aurones at a concentration of 100 μM for 3 days. The tdTomato specific fluorescence intensity (FI) was measured daily. The inhibitory rate was calculated as: $100-\left(\frac{\text{FI}\,\text{of}\,M\,t\,b\,\text{treated}\,\text{with}\,\text{aurones}\,\text{at}\,\mathrm{D3}-\mathrm{FI}\,\text{of}\,M\,t\,b\,\text{treated}\,\text{with}\,\text{aurones}\,\text{at}\,\text{D}\,0}{\mathrm{FI}\,\mathrm{of}\,M\,t\,b\,\text{untreated}\,\text{control}\,\text{at}\,\mathrm{D3}-\mathrm{FI}\,\mathrm{of}\,M\,t\,b\,\text{untreated}\,\text{control}\,\text{at}\,\mathrm{D0}}\right)\times 100$. For the round-1 screening, we used a diverse library of 87 aurones that consisted of a benzylidene, furanylidene, pyrrolylidene or thiophenylidene linked to benzofuranone with various bromine, chlorine, cyano, dimethylamino, fluorine, hydroxyl, iodine, methoxy, methyl, nitro, pyridyl or trifluoromethyl substitutions in benzylidene, furanylidene, pyrrolylidene or thiophenylidene. Some of the aurones also included bromine, hydroxyl, methoxy or methyl substitutions in benzofuranone. In general, these aurones were not effective inhibitors of *Mtb* growth. The three most effective aurones, 9067, 9251, and 9087, had inhibitory rates of 44.80%, 41.03% and 35.81%, respectively (Figure 1). The inhibitory rates of all the round-1 aurones on *Mtb* growth are shown in Supplementary Table S1. Based on the results of the first round of screening, another 43 aurones (round-2A) were synthesized to investigate additional substitutions of benzylidene in aurones. In these aurones, benzylidene was linked to benzofuranone and alternative benzofuranone groups, where the internal single bonded oxygen of benzofuranone was replaced with a nitrogen to generate azaaurones, an acetylated nitrogen to generate acetylated azaarones, a sulfur to generate thioaurones, or a carbonyl group to generate indanediones. The two most effective azaaurones, AA2A and AA8 had inhibitory rates of 85.95 and 71.86%, respectively (Figure 1). In round-2B of screening, another 16 aurones were synthesized to investigate additional substitutions in benzofuranone and the effect of glycosylation. The four most effective aurones, 9504, 9501, 9505, and 9510, had inhibitory rates of 88.29, 84.99, 84.44, and 83.14%, respectively (Figure 1). The inhibitory rates of all the round-2 aurones are shown in Table 1. The structures of all the aurones screened in this study are shown in Supplementary Table S2.

**TABLE 1:** Inhibitory rate of the round-2A and 2B aurones.

**Round-2A**				**Round-2B**	
**Aurone**	**Inhibitory rate**	**Aurone**	**Inhibitory rate**	**Aurone**	**Inhibitory rate**
3001	15.99 ± 4.90	11011	11.95 ± 2.02	9499	67.55 ± 5.73
3002	0.00	11012	37.56 ± 1.56	9501	84.99 ± 2.24
3004	10.45 ± 6.38	11014	28.54 ± 12.85	9503	38.82 ± 8.54
3005	0.00	AA10	17.60 ± 13.14	9504	88.29 ± 0.16
3006	0.00	AA11	43.38 ± 7.55	9505	84.44 ± 2.99
3007	29.27 ± 5.19	AA12	0.00	9506	50.14 ± 13.44
3008	13.17 ± 16.17	AA1A	26.24 ± 7.26	9507	56.30 ± 16.04
3009	27.50 ± 6.50	AA2	0.00	9510	83.14 ± 1.62
3011	17.74 ± 7.25	AA2A	85.95 ± 0.38	9512	63.03 ± 7.49
3012	69.28 ± 7.08*	AA3	67.28 ± 1.84	AK01	0.00
9260	22.25 ± 10.90	AA3A	41.32 ± 9.99	AK02	0.00
9301	17.08 ± 2.78	AA4A	66.18 ± 0.98	AK17	0.00
9304	14.77 ± 6.36	AA5	68.42 ± 0.51*	AK18	50.13 ± 9.23
9305	30.17 ± 2.66	AA5A	69.93 ± 10.09*	AK22	0.00
9312	44.83 ± 0.62	AA6	46.19 ± 1.70	AK30	9.18 ± 0.99
11001	22.57 ± 4.02	AA7	42.96 ± 2.20	AK33	24.50 ± 7.63
11003	35.80 ± 1.66	AA8	71.86 ± 2.00		
11004	30.81 ± 0.79	AA9	52.03 ± 1.05		
11005	13.22 ± 8.42	TA1	0.00		
11006	20.47 ± 3.90	TA2	0.00		
11007	29.60 ± 2.47	TA3	0.00		
11010	22.82 ± 2.45				

Inhibitory rate = 100 – (F.I. of treated Mtb at day-3 – F.I. of treated Mtb at day-0)/(F.I. of untreated Mtb at day-3 – F.I. of untreated Mtb at day-0) X 100. * Aurones 3012, AA5, and AA5A were highly fluorescent at the wavelength used to detect the tdTomato protein. For these aurones the inhibitory rate was determined using cellular absorbance. The inhibition rates are rounded to the second decimal place. Data are presented as Mean ± SD.

### Minimum Inhibitory Concentrations (MICs) and Cytotoxicity of the Six Aurone Leads

We determined MICs of the top six aurone leads (AA2A, AA8, 9501, 9504, 9505, and 9510) using the standard resazurin microtiter assay (REMA) (Palomino et al., 2002). The MIC was defined as the lowest drug concentration that prevented the color change of resazurin from blue to pink. The MICs for 9504, 9505, 9501, 9510, AA2A, and AA8 were 6.25, 12.5, 25, 25, 25, and 50 μM, respectively (Table 2). We evaluated the cytotoxic effects of aurones to human liver cells HepG2, primate kidney Vero cells and human monocyte THP-1 cells after a 2-days incubation using the microculture tetrazolium (MTT) cell viability assay. Concentration gradients of the selected aurones or RIF (as a control) were incubated with the three cell lines. We calculated half maximal inhibitory concentration (IC_50_) of aurones on these three cell lines. By comparing MICs with IC_50_, we calculated the selectivity index (SI) for each aurone lead as SI = MIC/IC_50_. Aurone 9504 had a SI higher than that of RIF on all of the cell lines. Aurones 9501 and 9510 have SIs > 10 on these three cell lines, which are comparable to the SI of RIF on two of the three cell lines (Table 2).

**TABLE 2:** Inhibition rates and MICs of the aurone leads against *Mtb*, IC_50_s of the leads against liver, kidney and monocytes, and SIs based on MICs and IC_50_s.

**Aurone and control**	**Inhibitory rate**	**MIC (μM)**	**IC_50_ (μM)**			**SI (IC_50_/MIC)**		
			**HepG2**	**Vero**	**THP-1**	**HepG2**	**Vero**	**THP-1**
AA2A	85.95 ± 0.38	25	117.81	128.28	109.78	4.71	5.13	4.39
AA8	71.86 ± 2.00	50	399.33	278.92	503.99	7.99	5.58	10.08
9501	84.99 ± 2.24	25	396.26	1, 112.47	324.64	15.85	44.50	12.98
9504	88.28 ± 0.16	6.25	790.14	549.85	628.13	126.40	87.98	100.50
9505	84.44 ± 2.99	12.5	29.28	479.26	219.46	2.34	38.34	17.56
9510	83.14 ± 1.62	25	451.46	892.38	692.70	18.06	35.69	27.71
RIF		0.5	4.56	22.78	15.03	9.12	45.56	30.06

### Aurone Leads Significantly Inhibit the Replication of Intracellular *Mtb*

**FIGURE 2:**
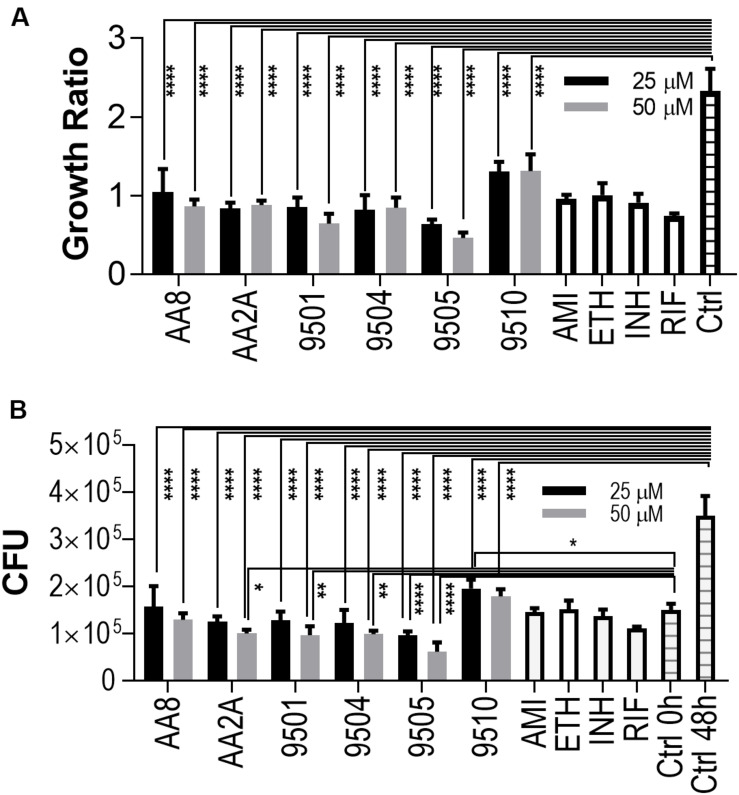
Aurones inhibit intracellular *Mtb*. **(A)** Growth ratios of samples treated with the aurone leads or control drugs compared to the growth ratio of untreated control. Growth ratio of each sample = FI of each sample at 48 h / FI of the same sample at 0 h. **(B)** CFUs of samples treated with the aurone leads or control drugs compared to the CFU of untreated control at 0 and 48 h. 25 and 50 μM of the aurone leads were incubated with the infected cells (MOI = 20) after removing extracellular bacteria. Positive controls: AMI (1 μg/mL), ETH (0.5 μg/mL), INH (0.5 μg/mL), and RIF (0.4 μg/mL). Data are means of three independent experiments ± S.D. *P < 0.05, **P < 0.01, ****P < 0.0001.

The intracellular activities of the six aurones were evaluated in the human macrophage THP-1 cell line. Amikacin, ethambutol, INH, and RIF were employed as positive controls at their *in vitro* MICs against intracellular *Mtb* (Brennan and Young, 2008). Twenty fiveand Fifty50 micrometer of all selected aurones except for 9510 completely inhibited intracellular *Mtb* replication (Growth ratio ~1) after 48 h of treatment (Figure 2A). The growth ratios of intracellular *Mtb* treated by the six aurones were all significantly lower than the untreated control (*P* < 0.0001) after the 48 h treatment. At 48 h, the CFUs of intracellular *Mtb* treated by the six aurones were all significantly lower than the untreated control (*P* < 0.0001). Compared to the intracellular CFU before the treatment started (0 h), the CFUs of samples treated by aurones 9501, 9504, 9505, AA2A, and AA8 at 48 h did not increase, suggest a bacteriostatic effect (Figure 2B). AA2A, 9501, 9504 at 50 μM and 9505 at both 25 and 50 μM reduced *Mtb* CFU significantly compared to the initial intracellular *Mtb* load (0 h). 9510 at 25 μM did not fully inhibit intracellular *Mtb* replication at 48 h.

### AA2A, AA8, 9501, and 9504 Can Significantly Reduce the Bacterial Load in the Lungs of *Mtb*-Infected Mice

**FIGURE 3:**
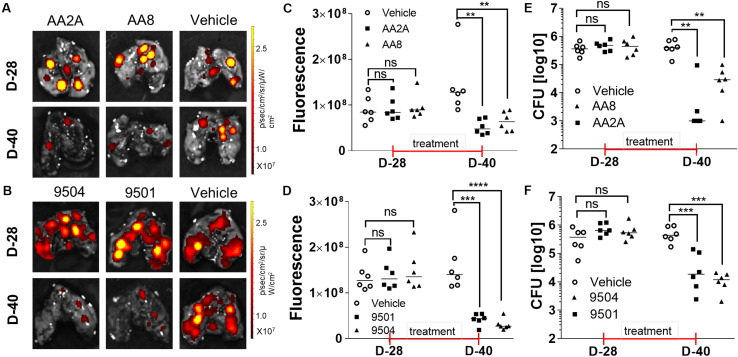
Evaluation of the efficacy of AA2A, AA8, 9501, and 9504 against *Mtb in vivo*. IVIS images of the mouse lungs treated with aurones AA2A or AA8 **(A)**, or with 9504 or 9501 **(B)**. Quantitative analysis IVIS *ex vivo* imaging results of the mouse lungs treated with aurones AA2A or AA8 **(C)**, or with 9504 or 9501 **(D)**. CFU in the lungs of infected mice treated with AA2A or AA8 **(E)**, or with 9501 or 9504 **(F)**. **P < 0.01; ***P < 0.001; ****P < 0.0001.

Mouse models have been widely used to evaluate efficacy of anti-TB drugs (Vandamme, 2014). Mouse strains with different genotypes vary in susceptibility to virulent *Mtb* (Medina and North, 1998). BALB/c mice are more susceptible to *Mtb* infection than other mice with increasing bacterial load and shorter survival time post-infection (Franzblau et al., 2012). Aerosol infection of *Mtb* is a widely accepted route of infection in the evaluation of candidate anti-TB drugs in mice (Orme, 1988). We determined *in vivo* efficacies of selected aurones against *Mtb* in aerosol-infected BALB/c mice. We selected two azaaurone leads and two aurone leads that had high SIs on the three cell lines in the *in vivo* efficacy evaluation. Prior to the evaluation of the aurones’ efficacies *in vivo*, we conducted a pilot drug-tolerance-test with four concentrations (1, 2, 5, 10 mg/kg/day) of the aurones. No acute toxic effects were identified at all tested doses for all the four aurones over the 7-days test. Because 5 mg/kg/day of the tested aurones appeared to be a safe dose for mice, we used this dose to treat infected mice thereafter. BALB/c mice were aerosol-infected by the tdTomato-expressing *Mtb* strain. At day-28 post-infection, mice were randomly grouped into the vehicle-, AA2A-, AA8-, 9501-, or 9504-treated groups. An IVIS Spectrum *in vivo* imaging system (PerkinElmer) was used to collected *ex vivo* images for the harvested lungs 1-day before the treatment started and on day-12 post-treatment, following the protocol described previously (Kong et al., 2016; Figures 3A,B). The quantitative IVIS imaging data showed that tdTomato specific FI of the treated groups of mice were significantly lower than that of the untreated mice (Figures 3C,D). Bacterial CFU counts in the lungs for each group of mice were also collected by plating homogenized lungs on agar plates. Compared to the untreated mice, the bacterial load of aurone-treated mice were reduced 1.32-2.22 log_10_ (2.22-log_10_ for AA2A, 1.67-log_10_ for 9504, 1.34-log_10_ for 9501, and 1.32-log_10_ for AA8) (Figures 3E,F).

### The Aurone Leads Inhibit *Mtb* Chorismate Synthase (Cs)

**FIGURE 4:**
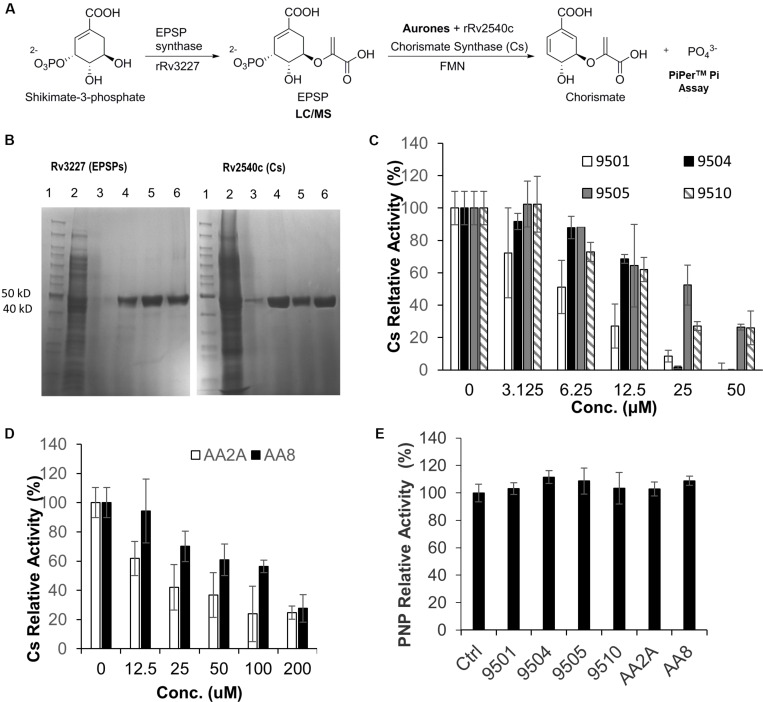
AA2A and 9504 inhibit *Mtb*-Cs activity. **(A)** Assay to measure the ability of aurones to inhibit Cs of *Mtb*. **(B)** SDS-PAGE analysis of extracted rRv3227 (left) and rRv2540c (right). Lane 1: marker (PageRuler^M^ Unstained); 2: crude protein extracts; 3–5: samples eluted from Ni-column by 125, 250, and 500 mM imidazole, respectively; 6: the concentrated proteins eluted by 500 mM imidazole. **(C)** Evaluation of 9501, 9504, 9505, and 9510 inhibitory effects on *Mtb*-Cs activity. **(D)** Evaluation of AA2A and AA8 inhibitory effects on *Mtb*-Cs activity. Data are means of three independent experiments ± S.D. The reaction mixture comprised *Mtb*-Cs, various concentrations of aurone leads, EPSP (15 μL), FMN (0.04 mM) and NADH (0.3 mM). The production of chorismate from the enzymatic reaction was determined by measuring Pi production using MESG (0.2 mM) and PNP (1 U). **(E)** The six aurone leads do not inhibit purine nucleoside phosphorylase (PNP). The reaction mixture comprised PNP (0.2 U), MESG (50 μM), potassium phosphate (10 mM), and aurones (50 μM for 9501, 9504, 9505, and 9510; 200 μM for AA2A and AA8). No significant differences (*P* > 0.05) were identified between each of the aurone-treated group and the positive control group. Data are means of three independent experiments ± S.D.

A derivative of benzofuran-3[2H]-one has been reported to inhibit the Cs of *S. pneumonia* (Thomas et al., 2003). It has a similar chemical structure to the six aurone leads in this study. In *Mtb*, the shikimate pathway is essential (Parish and Stoker, 2002) and leads to the biosynthesis of a wide range of primary and secondary metabolites, including aromatic amino acids, folate, naphthoquinones, menaquinones and mycobactins. *Mtb*-Cs converts 5-enol-pyruvyl shikimate-3-phosphate (EPSP) to chorismate via a 1,4-*trans* elimination of phosphate (Macheroux et al., 1999). The reaction requires a reduced flavin mononucleotide (FMNred) and NADH (Figure 4A). The *Mtb-*Cs serves as a NADH:FMN oxidoreductase in this reaction (Ely et al., 2008). We have constructed two plasmids expressing the *Mtb*-EPSP synthase (*Mtb*-EPSPs) (Rv3227) and *Mtb*-Cs (Rv2540c), respectively. We then extracted *Mtb*-EPSPs and *Mtb*-Cs from the soluble components of the lysates of the *E. coli* strains expressing them by affinity chromatography on a HisTrap HP Ni2+ IMAC column eluted with concentration gradient of imidazole. The samples were analyzed by SDS-PAGE, and the results showed that rRv2540c and rRv3227 had molecular masses as predicted (42.9 and 46.3 kD) based on their amino acid sequences (Figure 4B). We first synthesiaed EPSP using EPSPs, shikimate, and phosphoenolpyruvate and confirmed the generation of EPSP by LC/MS (Supplementary Figure S1) as described in materials and methods. We have analyzed the inhibitory effects of the aurone leads on *Mtb*-Cs activity by comparing Pi released from the chorismate synthesis reaction between samples treated with and without aurones, following the protocol described previously (Ely et al., 2008) with minor modifications as described in materials and methods. The production of chorismate from the enzymatic reaction was determined by measuring Pi production using the purine nucleoside phosphorylase (PNP) and 2-amino-6-mercapto-7-methylpurine ribonucleoside (MESG). The free Pi from the sample without Cs was measured as a background absorbance and subtracted from the absorbance of the aurone-treated and untreated samples. The untreated sample was added to the aurone dilution buffer containing the same concentration of DMSO as those of the aurone-treated samples and served as a positive control (activity 100%). We found that the samples co-incubated with the aurone leads showed significant Pi reduction compared to the untreated control with a dose-response effect, indicating that the aurone leads can inhibit *Mtb*-Cs activity (Figures 4C,D). We calculated the half maximal inhibitory concentration (IC_50_; μM) of aurones on *Mtb*-Cs activity as: 9501 (5.36 ± 1.65); 9504 (17.13 ± 1.07); 9505 (24.47 ± 9.44); 9510 (15.12 ± 1.68); AA2A (24.09 ± 0.55); and AA8 (50.3 ± 8.61). As the chorismate synthesis reaction involves another enzyme, PNP, we also ruled out the possibility that the observed Pi reduction of aurone-treated samples were derived from the inhibitory effects of the aurone leads on PNP (Figure 4E).

## Discussion

Aurones are a sub-family of the flavonoids. They feature the typical 15-carbon skeleton, but can be simply dissected into two main parts—a benzofuranone and an exocyclic arylidene that is most frequently derived from an aldehyde. While known for some time, it is only more recently that the biological activity of aurones has been a topic of active interest (Popova et al., 2019). Previously, a broad-spectrum kinase inhibitor screening have identified three aurones having activity against *Mtb* (Reynolds et al., 2012). All of the three aurones featured an ester tethered to the aurone scaffold via a phenolic ether linkage. While active and fairly selective, the probable cellular target as well as more complete SAR results were not investigated.

The aurone ring system remains underexplored, particularly compared to other members of the flavonoid family of natural products. A related system, the azaaurones in which the ring oxygen is replaced by a nitrogen, has been the subject of much less study and yet may possess certain advantages. In this study, an iterative two-round strategy was utilized to generate six aurone compounds, 9504, 9505, 9501, 9510, AA2A, and AA8, that could effectively inhibit the growth of *Mtb* in culture and intracellularly in human cells. An assessment of the structures of the most inhibitory aurones from the two rounds of testing demonstrated some interesting trends in what constitutes the design of aurones that are effective in inhibiting *Mtb*. In general the most inhibitory aurones were aurones where benzylidene with dimethylamino substitutions were linked to benzofuranone with chlorine or bromine substitutions and acetylated or non-acetylated azaaurones where benzylidene was linked to indolin-3-one. These aurone scaffolds represent excellent platforms for the development of future anti-TB compounds. 6-chloro, 6-bromo, 7-bromo, and 5,6-dimethyl appeared to be the optimal substitution patterns on the benzofuranone portion and the modification of the dimethylamino group was not well tolerated. Based on the six leads, we have designed more derivatives, such as iodo, methyl, ethyl, and methoxy substitutions at the 5, 6, and 7 position of benzofuranone portion. Further characterization of their efficacies *in vitro* and *in vivo* will lead to identification of more active, safer, and orally bioavailable aurone analogs.

First-line anti-TB drugs contribute to diverse pathological complications, and hepatotoxicity is one of them. Current first-line anti-TB drugs are amongst the most reported anti-microbial drugs incriminated to be potential causes of drug-induced liver injury (Pugh et al., 2009), and anti-TB drug induced liver injury is one of the most prevalent hepatotoxicities reported in many countries (Huang, 2014). When TB patients take these drugs, liver function has to be followed every 2 weeks to prevent serious hepatotoxicity. Sometimes drugs have to be stopped until liver functions improve. The 9504, 9510, AA8, and 9501 had low cytotoxic effects on the human liver cell line HepG-2 and the primate kidney cell Vero. Among them 9504 had the highest selectivity index on both cells, which is significantly better than RIF, suggesting that it could be an effective replacement for the more toxic anti-TB drugs that are currently in use. However, AA2A and 9505 did show some degree of cytotoxicity on the cell lines. We will design and synthesize more derivatives of these aurones to identify the low cytotoxic compounds for further efficacy evaluation.

Because *Mtb* primarily stays in the macrophage after infection, it is critical to evaluate efficacies of new chemicals against intracellular *Mtb*. A few aurone analogs have been reported to actively inhibit an intracellular parasite, *Leishmania infantum*, in THP-1 cells (Roussaki et al., 2012). We found that the six aurone leads could significantly inhibit intracellular *Mtb* replication at two concentrations with no or minimal cytotoxic effect. These data indicate that aurone leads can effectively penetrate into the macrophage phagosome to inhibit *Mtb* replication. Furthermore, our *in vivo* studies of 9504, 9501, AA2A, and AA8 demonstrated that these four aurones at 5 mg/kg could rapidly reduce more than 1-log_10_ CFU of *Mtb* in mouse lungs. Using 10 mg/kg of RIF with 12 daily oral doses, Jayaram et al. have demonstrated a ∼1-log_10_ CFU reduction in bacterial burden in the lungs (Jayaram et al., 2003). These data suggest that the *in vivo* efficacy of the tested aurone leads are comparable to RIF. Interestingly, the MICs of the aurone leads are greater than RIF’s MIC. We speculate that the relatively high *in vivo* efficacy of these leads are due to their high stability and/or long retention time *in vivo*. Detailed absorption, distribution, metabolism, and excretion (ADME) and pharmacokinetic (PK) and pharmacodynamic (PD) studies are planned to examine this hypothesis.

A derivative of benzofuran-3[2H]-one, which inhibits the chorismate synthase (Cs) of *S. pneumonia* (10), has a similar chemical structure to the top six aurones isolated in this study that were inhibitory to *Mtb*. In *Mtb*, the shikimate pathway is essential (Parish and Stoker, 2002) and the *Mtb*-Cs is the key enzyme for the last step of the pathway. Chorismate, the final product of the shikimate pathway is required for the synthesis of aromatic amino acids, folate, naphthoquinones, menaquinones and mycobactins (Parish and Stoker, 2002). Because the shikimate pathway of *Mtb* is essential and is absent from mammals (Parish and Stoker, 2002; Ahn et al., 2004), *Mtb*-Cs is an attractive drug target since inhibition of Cs is unlikely to have a toxic side effect on the host. Although our data demonstrated that the six aurone leads can significantly inhibit *Mtb*-Cs, the relatively high concentrations required for inhibition of Cs suggest that Cs might not be the only target for the aurone leads. To verify this, we plan to overexpress Cs in *Mtb* and determine the MICs of the aurones against the Cs-overexpressed strain, and also examine whether the inhibition of *Mtb* by these aurones can be rescued by supplementation of down-stream products, e.g., aromatic amino acids. We will also obtain revertants that are resistant to the aurone leads, and subject the revertants to whole genome sequencing to further explore the mechanism of action.

## Data Availability Statement

All datasets generated for this study are included in the article/Supplementary Material.

## Ethics Statement

The animal study was reviewed and approved by The UTHSC Institutional Animal Care and Use Committee.

## Author Contributions

DY, SH, JL, CJ, EA, and YK conceived and designed the experiments. DY, SL, ZT, JL, JS, LZ, and YK performed the experiments. DY, SL, JL, SH, EA, and YK analyzed the data. SH, EA, JL, CJ, JS, and YK contributed reagents, materials, and analysis tools. DY, SH, EA, and YK wrote the manuscript.

## Conflict of Interest

EA has equity ownership in GreenWay Herbal Products, LLC. The remaining authors declare that the research was conducted in the absence of any commercial or financial relationships that could be construed as a potential conflict of interest.

## Supplementary Material

FIGURE S1 Detect EPSP by LC/MS in samples after the assay of EPSP synthesis. One sample (Cs –) was collected right after the EPSP synthesis reaction and before chorismate synthesis. Three samples (Cs +) were collected immediately after the chorismate synthesis reaction. EPSP eluted with a retention time of 1.3 min and the characteristic peak of EPSP was ESI m/z 323.2 [M-1]- under the negative ESI mode. The peak areas (AUC) of EPSP were calculated from extracted ion chromatograms (EIC) of ESI- m/z 323.2 ± 0.5.

TABLE S1 Inhibitory rates of the round 1 aurones on *Mtb* growth at 100 μM.

TABLE S2 Structure of the aurones used in this study.
