# Supplementary figures and images for: Identification of Anti-tuberculosis Compounds From Aurone Analogs
Source: Front Microbiol. 2020 May 20;11:1004. doi: 10.3389/fmicb.2020.01004 (PMC7251074; doi:10.3389/fmicb.2020.01004)

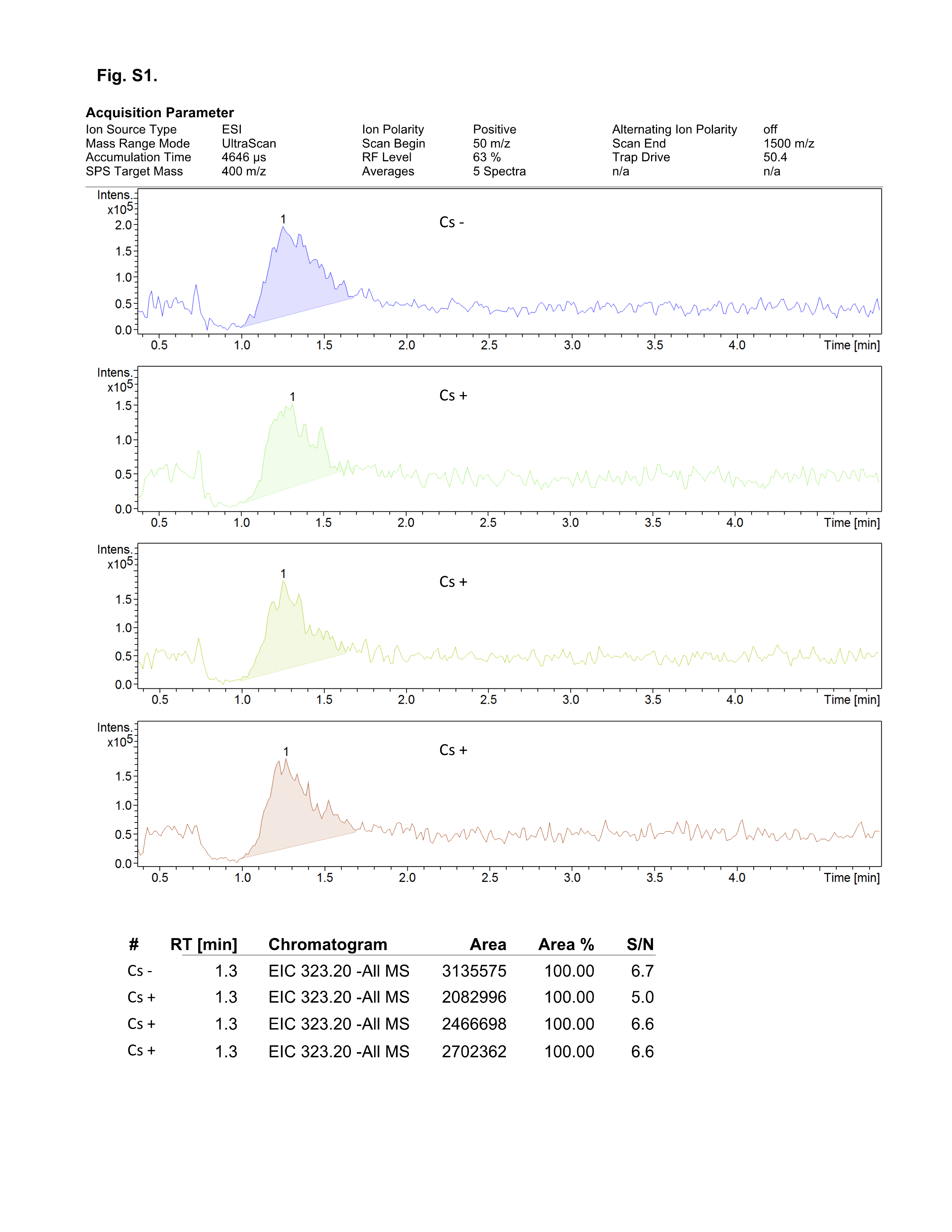

Supplement: FIGURE S1 — Detect EPSP by LC/MS in samples after the assay of EPSP synthesis. One sample (Cs –) was collected right after the EPSP synthesis reaction and before chorismate synthesis. Three samples (Cs +) were collected immediately after the chorismate synthesis reaction. EPSP eluted with a retention time of 1.3 min and the characteristic peak of EPSP was ESI m/z 323.2 [M-1]- under the negative ESI mode. The peak areas (AUC) of EPSP were calculated from extracted ion chromatograms (EIC) of ESI- m/z 323.2 ± 0.5. [file Image_1.tif]

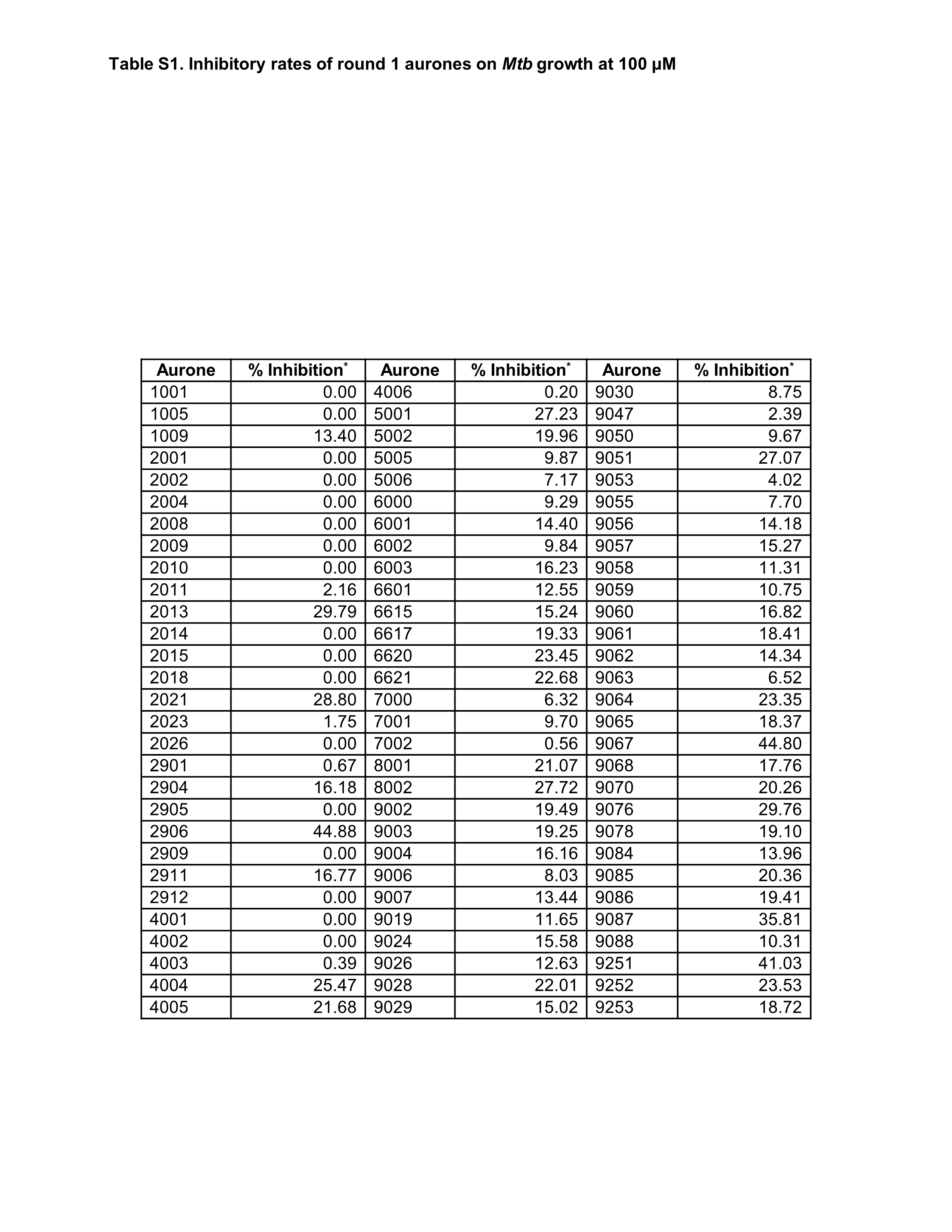

Supplement: TABLE S1 — Inhibitory rates of the round 1 aurones on Mtb growth at 100 μM. [file Data_Sheet_1.zip › Table S1.TIF]
